# The neutralizing function of the anti-HTLV-1 antibody is essential in preventing in vivo transmission of HTLV-1 to human T cells in NOD-SCID/γcnull (NOG) mice

**DOI:** 10.1186/s12977-014-0074-z

**Published:** 2014-08-28

**Authors:** Mineki Saito, Reiko Tanaka, Hideki Fujii, Akira Kodama, Yoshiaki Takahashi, Toshio Matsuzaki, Hiroshi Takashima, Yuetsu Tanaka

**Affiliations:** Department of Immunology, Graduate School of Medicine, University of the Ryukyus, 207 Uehara, Okinawa, 903-0215 Japan; Department of Microbiology, Kawasaki Medical School, 577 Matsushima, Kurashiki, Okayama 701-0192 Japan; Department of Neurology and Geriatrics, Kagoshima University Graduate School of Medical and Dental Sciences, 8-35-1 Sakuragaoka, Kagoshima, 890-8520 Japan

**Keywords:** HTLV-1, NOG mice, Intrasplenic injection, Neutralizing antibody, Envelope gp46

## Abstract

**Background:**

Human T-cell leukemia virus type 1 (HTLV-1) causes both neoplastic and inflammatory diseases, including adult T-cell leukemia and HTLV-1-associated myelopathy/tropical spastic paraparesis (HAM/TSP). Because these life-threatening and disabling diseases are not yet curable, it is important to prevent new HTLV-1 infections.

**Findings:**

In this study, we have established a simple humanized mouse model of HTLV-1 infection for evaluating prophylactic and therapeutic interventions. In this model, HTLV-1-negative normal human peripheral blood mononuclear cells (PBMCs) are transplanted directly into the spleens of severely immunodeficient NOD-SCID/γcnull (NOG) mice, together with mitomycin-treated HTLV-1-producing T cells. Using this model, we tested the efficacy of monoclonal antibodies (mAbs) specific to HTLV-1 as well as human IgG isolated from HAM/TSP patients (HAM-IgG) in preventing HTLV-1-infection. One hour before and 24 h after transplantation of the human cells, each antibody sample was inoculated intraperitoneally. On day 14, human PBMCs isolated from the mouse spleens were tested for HTLV-1 infection. Whereas fresh CD4-positive and CD8-positive T cells isolated from untreated mice or mice treated with isotype control mAb, HTLV-1 non-neutralizing mAbs to envelope gp46, gag p19, and normal human IgG were all infected with HTLV-1; the mice treated with either HTLV-1 neutralizing anti-gp46 mAb or HAM-IgG did not become infected.

**Conclusions:**

Our data indicate that the neutralizing function of the antibody, but not the antigen specificity, is essential for preventing the *in vivo* transmission of HTLV-1. The present animal model will also be useful for the *in vivo* evaluation of the efficacy of candidate molecules to be used as prophylactic and therapeutic intervention against HTLV-1 infection.

**Electronic supplementary material:**

The online version of this article (doi:10.1186/s12977-014-0074-z) contains supplementary material, which is available to authorized users.

## Findings

Human T cell leukemia virus type-1 (HTLV-1) has been linked to the development of adult T-cell leukemia (ATL) and a chronic inflammatory disease called HTLV-1-associated myelopathy/tropical spastic paraparesis (HAM/TSP) [1]. However, the mechanism of disease pathophysiology is still incompletely understood, and the treatments available are still unsatisfactory. Therefore, studies should be conducted to develop an effective method for preventing the occurrence of new infections, as well as to identify the mechanism of disease development and effective treatment following infection. This will require the development of a small animal model that can be exploited as a tool for the screening and evaluation of HTLV-1 infection. However, although HTLV-1 consistently infects rabbits [2,3], some non-human primates [4,5], and to a lesser extent, rats [6,7], the virus does not efficiently infect murine cells. Previous studies have indicated that viral transmission in mice, using typical methods of infection, results in inconsistent infections and limited virus expression in tissues [8-10].

Here we established a novel mouse model to evaluate primary HTLV-1 infection of human lymphocytes *in vivo*. In this model, HTLV-1-negative healthy human peripheral blood mononuclear cells (PBMCs) (2 × 10^6^/mouse) were transplanted directly into the spleens of severely immunodeficient NOD-SCID/γcnull (NOG) mice, together with cells from the mitomycin C (MMC)-treated HTLV-1-infected cell line ILT-M1 (1 × 10^6^/mouse), which is an IL-2-dependent CD8+ T cell line derived from a HAM/TSP patient (kindly provided by Prof. Kannagi of Tokyo Medical and Dental University). Cell suspensions in a final volume of 50 μl were administered by intrasplenic injection (hereafter called hu-PBMC-NOG-spl mice). As previously reported [11], the severe immune deficiency of the NOG strain enables efficient engraftment of the human T cells, and a reduction in mouse death caused by severe graft-versus-host disease (GVHD), compared to those inoculated into the peritoneal cavity, which is the more common route of administration. In fact, all mice grew normally without piloerection or weight loss until 14 days after transplantation (i.e., the time of sacrifice).

**Figure 1:**
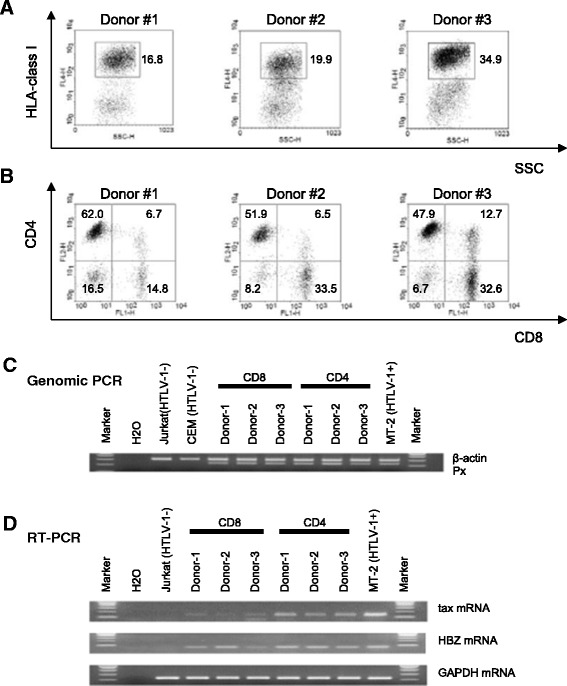
**In vivo infection of HTLV-1 in engrafted human PBMCs in hu**-**PBMC**-**NOG**-**spl mice. A**. Live cells were gated on their forward and side light scatter characteristics, and then cell surface markers within the HLA-class I-positive population were analyzed. **B**. There tended to be higher frequencies of CD4-positive cells than CD8-positive cells. The numbers represent the percentage of the cell population within the HLA-class I-positive gate. **C**. Genomic PCR to confirm HTLV-1 infection. Genomic DNA was extracted from human CD4 and CD8-positive T cells recovered from the spleens of hu-PBMC-NOG-spl mice sacrificed 14 days post infection, and then a fragment of the HTLV-1 pX region was amplified. β-actin was used as a control. The lower limit of detection was one copy of HTLV-1 tax per 10^4^ PBMCs. **D**. RT-PCR to confirm HTLV-1 infection. RNA was extracted from human CD4 and CD8-positive T cells recovered from the spleens of hu-PBMC-NOG-spl mice sacrificed 14 days post infection. cDNA was synthesized and amplified from HTLV-1 tax and the HBZ region as described previously [15]. GAPDH was used as a control.

First, we isolated the bulk spleen cells from hu-PBMC-NOG-spl mice sacrificed 14 days post inoculation/infection. Using flow cytometry (FCM), live cells were gated on their forward and side light scatter characteristics, and cell surface markers within the HLA-class I-positive population (i.e., human cells) were analyzed (Figure 1A). The numbers of recovered human cells (i.e. HLA-class I positive cells) from the mouse spleens were 1.48 × 10^7^ (Donor #1), 1.29 × 10^7^ (Donor #2) and 1.92 × 10^7^ (Donor #3), respectively, which are much higher than the numbers of inoculated human cells, suggesting successful engraftment. The increased numbers of human T cells in the mouse spleens within two weeks after inoculation may have been caused by xenoreactive lymphocyte proliferation, since recent report by Søndergaard et al. suggested that injection of human PBMCs into NOG mice cause polyclonal expansion and activation of functional human T cells [12]. Meanwhile, human T cell expansion due to HTLV-1 is unlikely, since there is no clear difference in numbers of human T cells in the mouse spleens between mice treated with PBS (i.e., HTLV-1-infected) and mice treated with neutralizing antibodies (i.e., HTLV-1-uninfected) (data not shown). There tended to be higher frequencies of CD4-positive cells than CD8-positive cells (Figure 1B). Next, in order to confirm HTLV-1 infection, we isolated human CD4- and CD8-positive T cells by positive immunoselection from the bulk spleen cells, and then amplified a fragment of the HTLV-1 pX region by genomic PCR (Figure 1C). As shown in Figure 1C, similar to the naturally HTLV-1-infected PBMCs from healthy carriers and HAM/TSP patients, an HTLV-1 proviral DNA band was detected in all the isolated human CD4- and CD8-positive cell samples tested. We also performed RT-PCR in order to detect viral mRNA (tax and HBZ) in these human CD4- and CD8-positive T cells. As shown, all of the CD4 and CD8 cells tested expressed both tax and HBZ mRNA (Figure 1D). The poor visibility of tax mRNA bands of CD8 cells suggest that the possible contamination of residual ILT-M1 cells, which are positive for CD8 and strongly express tax mRNA, is unlikely. To further rule out the possible contamination of residual ILT-M1 cells, inverse PCR amplification was carried out to determine the sequences adjacent to HTLV-1 LTRs (both 3′- and 5′-LTR) using the DNA extracted from ILT-M1 cells, as previously described [13]. Next, integration site-specific PCR was carried out using primer pairs that encompass HTLV-1 LTRs (both 3′- and 5′-LTR) and flanking host sequences (Additional file 1: Table S1). As shown in Additional file 2: Figure S1, no integration site-specific bands were observed except for ILT-M1 cells, suggesting that the possible contamination of HTLV-1 genome derived from the residual ILT-M1 cells is unlikely. The median proviral DNA copy numbers (proviral load: PVL) in 1 × 10^4^ of both the human CD4 and human CD8 cells recovered from three hu-PBMC-NOG-spl mice, each inoculated with human PBMCs from different donors, were 9,533 and 4,546, respectively (i.e., 0.95 and 0.45 copies/cell, respectively), suggesting highly efficient cell-to-cell transmission of HTLV-1 from infected to uninfected human lymphocytes *in vivo*. Although a previous study also showed the successful engraftment of an HTLV-1-transformed cell line and uninfected PBMCs in NOG mice, the HTLV-1 PVL in spleen was very low and less than 1% of cells were infected with HTLV-1 [14]. In this previous study, 10^7^ uninfected human PBMCs were injected intraperitoneally, and those PBMCs were infected with HTLV-1 by intraperitoneal inoculation of MMC-treated HTLV-1-infected MT-2 cells (10^3^ or 10^4^ cells/mouse) [14]. The different infection efficiencies between the previous and present studies clearly indicate that the efficient engraftment of the human T cells *in vivo* could be achieved by this route of inoculation. As shown in the present study, an intrasplenic transfer of human PBMCs can reduce the number of PBMCs required for the initial inoculation by approximately 1 log unit for the generation of more than 10^7^ human T cells within two weeks, probably because human lymphocytes directly inoculated into the mouse spleen are efficiently activated, and thus HTLV-1 could efficiently infect human T cells *in vivo*. The microanatomic environment of the secondary lymphoid organs, such as the spleen, might also play an important role in the efficient cell-to-cell transmission of HTLV-1.

**Figure 2:**
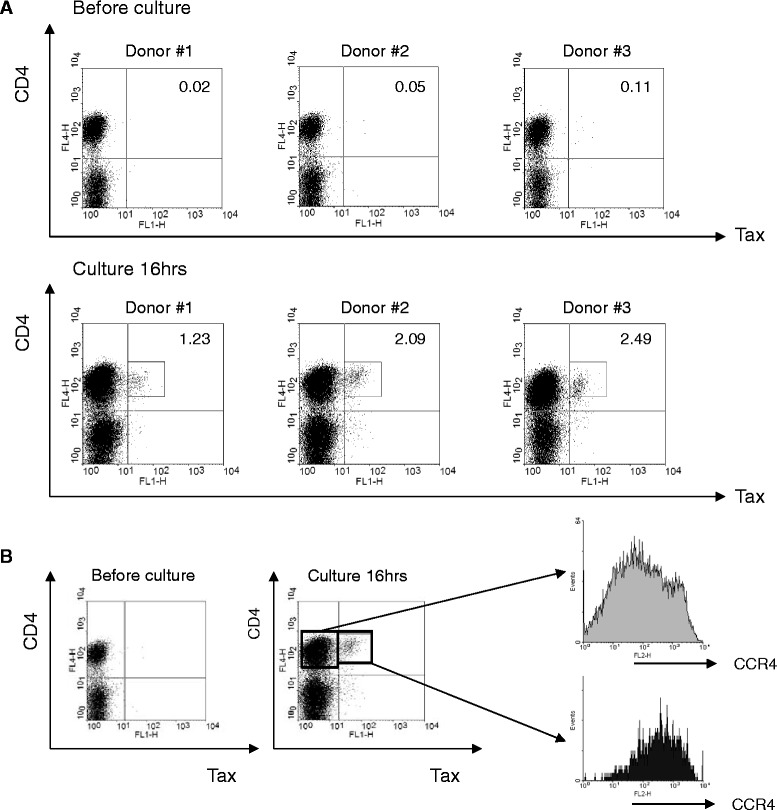
**Characteristics of HTLV**-**1**-**infected human T cells recovered from hu**-**PBMC**-**NOG**-**spl mice.** Tax protein expression in human lymphocytes recovered from the spleens of hu-PBMC-NOG-spl mice by flow cytometry. **A**. Human lymphocytes recovered from mouse spleens express very low levels of Tax protein (upper panel). After a short-term (16 h) cultivation ex vivo, Tax expression was rapidly induced (lower panel). The numbers represent the percentage of the Tax-positive cell population within the HLA-class I-positive gate. **B**. Tax-expressing cells are more frequently positive for CCR4 than Tax-negative cells.

It is well known that viral gene transcription of HTLV-1 in vivo is suppressed in the PBMCs of most HTLV-1-infected individuals [16]. To test whether this phenomenon occurs even in hu-PBMC-NOG-spl mice, we examined HTLV-1 transactivator Tax protein expression in fresh and cultured human lymphocytes recovered from the spleens of infected hu-PBMC-NOG-spl mice by FCM. Similar to naturally HTLV-1-infected cells from healthy carriers and HAM/TSP patients [16], the fresh human lymphocytes recovered from the mouse spleens expressed very low levels of Tax protein (Figure 2A, upper panel). However, Tax expression was rapidly induced after short-term (16 h) cultivation ex vivo (Figure 2A, lower panel). Furthermore, these Tax-expressing CD4-positive cells were more frequently positive for chemokine (C-C motif) receptor 4 (CCR4) than Tax-negative CD4-positive cells (Figure 2B), as previously reported in natural HTLV-1 infections [17,18]. However, although most of the CD4-positive T cells recovered from mouse spleens were infected with HTLV-1, the number of Tax positive cells after ex vivo culture appeared to be small. This observation might be attributed to the culture conditions of this experiment. Specifically, we cultured whole cells isolated from the recipient mouse spleens, indicating the mixed cultures of inoculated human PBMCs and mouse cells including stromal cells. Recently, Kinpara et al. reported that expression of HTLV-1 in HTLV-1-infected T cells is markedly suppressed at both the mRNA and protein levels upon co-culture of human cells and mouse stromal cells, in part via the type I interferon (IFN) response [19]. It is therefore plausible that the observed small number of Tax-expressing cells after ex vivo culture is likely due to co-culture with mouse stromal cells derived from the spleen. Meanwhile, we observed that the percentage of Tax-expressing cells in the same culture conditions varies from one patient to another even in HAM/TSP patients with similar PVL (Saito et al., unpublished data). Furthermore, the severely immune-deficient NOG mice used in this study do not have any acquired immune response against inoculated HTLV-1 infected cells, such as HTLV-1-specific Abs, helper T cells, and cytotoxic T lymphocytes. These observations suggest that not only culture conditions but also cellular factors might be involved in the number of Tax-expressing cells. It also needs to be clarified whether the small number of Tax-expressing cells can be explained by multiple infection of single cell. Further investigations of such factors would be important for controlling HTLV-1 infection and disease development in vivo.

**Figure 3:**
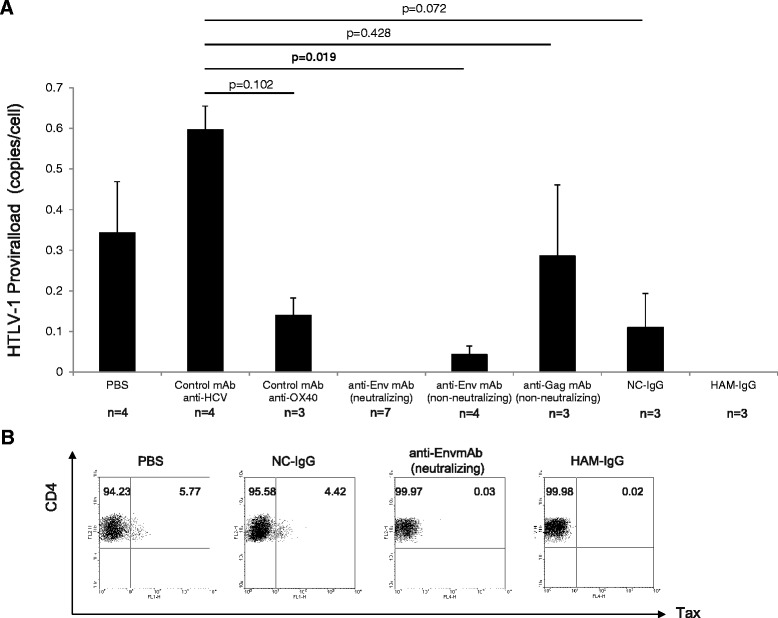
**HTLV-** **1 infection in hu-** **PBMC-** **NOG-** **spl mice was completely inhibited by neutralizing,** **but not non-** **neutralizing,** **antibodies.** *In vivo* transmission of HTLV-1 and protective efficacy of various monoclonal antibodies was evaluated using quantitative real-time PCR analysis of HTLV-1 proviral DNA. Genomic DNA was extracted from the human lymphocytes recovered from hu-PBMC-NOG-spl mice. **A**. All of the mice immunized with neutralizing mAbs against Env (clone LAT-27) were completely protected against HTLV-1 infection, whereas non-neutralizing mAbs against Env (clone LAT-25), anti-Gag (clone GIN-7), anti-HCV (clone MO-8), or anti-OX40 mAb (clone B-7B5) did not protect against infection. The mice immunized with human immunoglobulin isolated from HAM/TSP patients (HAM-IgG) were also protected against HTLV-1 infection, whereas human IgG isolated from normal uninfected controls (NC-IgG) did not protect against infection. Results are shown as mean ± SE. To test for significant differences among the different groups, one-way analysis of variance was performed, followed by Scheffe’s multiple comparisons test. The lower limit of detection was one copy of HTLV-1 tax per 10^4^ PBMCs. **B**. Flow cytometric studies indicated that the human lymphocytes recovered from mouse spleens immunized with anti-Env neutralizing mAbs or HAM-IgG express only a trace amount of Tax protein after short-term (16 h) cultivation *ex vivo*, which may be a background false-positive staining artifact. In contrast, a significant amount of Tax protein was expressed in human lymphocytes recovered from non-immunized mouse spleens (PBS-injected) or mouse spleens immunized with NC-IgG. The numbers represent the percentage of the cell population within the HLA-class I-positive/CD4-positive gate.

It is well established that the HTLV-1 virions are not very infectious, and thus cell-to-cell transmission is more efficient both *in vivo* and *in vitro* [20,21]. The surface glycoproteins of HTLV-1, which are recognized by neutralizing antibodies, play important roles in cell-to-cell transmission [22,23]. Indeed, previous reports have indicated that passive transfer of HTLV-1 Env-specific-neutralizing antibodies is effective in preventing *in vivo* infection in macaques [5,24] and rabbit [25,26] models. However, these studies evaluated the in vivo transmission of HTLV-1 to non-human cells, which are more resistant to HTLV-1 infection than human cells are. In this study, we tested the protective efficacy of various anti-HTLV-1 antibodies against HTLV-1 transmission into human lymphocytes *in vivo* in the hu-PBMC-NOG-spl mouse model. The mice immunized with the anti-HTLV-1 gp46 neutralizing mAb (clone LAT-27) were completely protected against HTLV-1 infection whereas other non-neutralizing antibodies such as anti-gp46 mAb (clone LAT-25), anti-Gag (clone GIN-7), anti-HCV (clone MO-8), and anti-OX40 mAb (clone B-7B5) did not protect against infection (Figure 3A). The HTLV-1 proviral DNA was not detected by quantitative real-time PCR in the human lymphocytes recovered from hu-PBMC-NOG-spl mice that received passive transfer of LAT-27, indicating that the neutralizing function is an essential factor in preventing in vivo HTLV-1 transmission. Furthermore, passive immunization with human polyclonal anti-HTLV-1 IgG from HAM/TSP patients (HAM-IgG) can also protect against HTLV-1 infection in vivo, whereas human immunoglobulin isolated from HTLV-1-negative donors (NC-IgG) did not (Figure 3A). Consistent with the results of the quantitative real-time PCR, FCM studies also showed that the human CD4-positive cells recovered from mouse spleens immunized with either LAT-27 or HAM-IgG, express only trace amounts of Tax protein after short-term (16 h) cultivation *ex vivo*, which may be the result of background false-positive staining. In contrast, a significant amount of Tax protein was expressed in human lymphocytes recovered from non-immunized mouse spleens (PBS-injected) or mouse spleens immunized with NC-IgG (Figure 3B). These results demonstrate the requirement for the neutralizing function of the anti-HTLV-1 antibody in preventing *in vivo* transmission. It is noteworthy that neutralizing anti-Env gp46 clone LAT-27 and HAM-IgG completely blocked the in vivo transmission of HTLV-1 in human lymphocytes, even in the conditions that permit the vigorous proliferation of human lymphocytes that enables HTLV-1 to rapidly spread by cell-to-cell contact. However, antibody injection only once after PBMC transplantation did not block the HTLV-1 infection in vivo, suggesting that the pre-existing neutralizing anti-Env Abs are critical for preventing HTLV-1 infection (Additional file 3: Figure S2). This result also suggests that *in vivo* transmission is established within 24 hours after transfer of HTLV-1-infected cells. Importantly, although neutralizing Abs used in this study displayed antibody-dependent cell-mediated cytotoxicity (ADCC) activity in vitro in our previous study [27], such neutralizing and ADCC activities of anti-Env Abs are not crucial for the elimination of HTLV-1-infected cells once HTLV-1 infection is established *in vivo*. Indeed, titers of existing neutralizing and ADCC Abs did not correlate with HTLV-1 PVL (i.e., numbers of HTLV-1-infected cells in vivo) (Saito et al., unpublished data). Moreover, HAM/TSP patients also showed high titers of such Abs, indicating that these Abs are not potent in preventing the onset of HAM/TSP in infected individuals. These data also indicate the importance of passive immunization before infection.

Recently, we reported that both LAT-27 and HAM-IgG, but not non-neutralizing LAT-25 and NC-IgG, are capable of depleting and/or eliminating HTLV-1-infected cells in the presence of autologous PBMCs in vitro. This occurs in part via ADCC, preventing the spontaneous immortalization of T cells [27]. Thus, the neutralizing activity is essential for preventing HTLV-1 infection as well as malignant transformation. In the present study, although non-neutralizing anti-Env gp46 (clone LAT-25) and anti-Gag p19 (clone GIN-7), as well as control antibodies (anti-HCV clone MO-8, anti-OX40 clone B-7B5), and normal human IgG (NC-IgG) did not completely block the infection, we observed that non-neutralizing LAT-25 mAb and anti-OX40 mAb decreased the number of HTLV-1 infected cells to some extent (Figure 3A). Since OX40 is a cell-surface molecule specifically expressed on HTLV-1-infected and activated T cells [28], and LAT-25 recognizes the HTLV-1 Env protein, these data may suggest a novel effect of IgG specifically reacting with a number of membrane receptors on HTLV-1-infected and/or activated T cells *in vivo*. As shown in Figure 3A, human IgGs isolated from uninfected people also suppressed the PVL, indicating that the administered non-specific IgGs also can help to eliminate the inoculated HTLV-1-infected cells (i.e., ILT-M1 cells). It is well established that the intravenous immunoglobulins (IVIg) therapy is effective in various diseases including autoimmune diseases and life-threatening infections. Although the precise mechanism of action of injected IVIg is not fully understood, several pathophysiological mechanisms such as suppression of idiotypic antibodies, saturation of Fc receptors on macrophages, modulation of complement activation, and suppression of various immunomodulators such as cytokines, chemokines, and metalloproteinases have been proposed [29]. It is therefore possible that the administered non-specific immunoglobulin in our mouse model also can help to eliminate the inoculated HTLV-1-infected cells (i.e. ILT-M1 cells) via unknown mechanisms, resulting in a decreased efficiency of *in vivo* infection.

In conclusion, we have established a novel and simple small animal model to study primary HTLV-1 infection in vivo. Although our mouse model is not the animal models of HAM/TSP or ATL, the present study has demonstrated an important rational basis for passive immunization against HTLV-1 infection in humans. Using our mouse model, *in vivo* evaluation of the efficacy of drug candidates could also be investigated in future studies.

## Additional files

Additional file 1: Table S1. Sequences flanking the integration site of HTLV-1 provirus in ILT-M1 cell and primer sequences used for integration site-specific PCR. Additional file 2: Figure S1. To rule out the possible contamination of residual ILT-M1 cells, inverse PCR amplification was carried out to determine the sequences adjacent to HTLV-1 LTRs (both 3′- and 5′-LTR) using the DNA extracted from ILT-M1 cells, as previously described [13]. Then, integration site-specific PCR was carried out using primer pairs that encompass HTLV-1 LTRs (both 3′ and 5′ LTR) and flanking host sequences (Additional file 1: Table S1). As shown, no integration site specific bands were observed except for ILT-M1 cells, suggesting that the possible contamination of HTLV-1 genome derived from the residual ILT-M1 cells is unlikely. Additional file 3: Figure S2. Flow cytometric studies showed that the human lymphocytes recovered from mouse spleens express the amount of Tax protein after short-term (16 h) cultivation ex vivo, indicating that the neutralizing anti-Env Ab (clone LAT-27) injection once after PBMC transplantation did not block the in vivo transmission of HTLV-1.

## Abbreviations

HTLV-1: Human T-cell leukemia virus type-1
ATL: Adult T-cell leukemia
HAM/TSP: HTLV-1-associated myelopathy/tropical spastic paraparesis
ADCC: Antibody-dependent cellular cytotoxicity
PVL: Proviral load

## References

[1] Uchiyama T (1997). Human T cell leukemia virus type I (HTLV-I) and human diseases. Annu Rev Immunol.

[2] Akagi T, Takeda I, Oka T, Ohtsuki Y, Yano S, Miyoshi I (1985). Experimental infection of rabbits with human T-cell leukemia virus type I. Jpn J Cancer Res.

[3] Lairmore MD, Roberts B, Frank D, Rovnak J, Weiser MG, Cockerell GL (1992). Comparative biological responses of rabbits infected with human T-lymphotropic virus type I isolates from patients with lymphoproliferative and neurodegenerative disease. Int J Cancer.

[4] Nakamura H, Hayami M, Ohta Y, Ishikawa K, Tsujimoto H, Kiyokawa T, Yoshida M, Sasagawa A, Honjo S (1987). Protection of cynomolgus monkeys against infection by human T-cell leukemia virus type-I by immunization with viral env gene products produced in Escherichia coli. Int J Cancer.

[5] Murata N, Hakoda E, Machida H, Ikezoe T, Sawada T, Hoshino H, Miyoshi I (1996). Prevention of human T cell lymphotropic virus type I infection in Japanese macaques by passive immunization. Leukemia.

[6] Suga T, Kameyama T, Kinoshita T, Shimotohno K, Matsumura M, Tanaka H, Kushida S, Ami Y, Uchida M, Uchida K, Miwa M (1991). Infection of rats with HTLV-1: a small-animal model for HTLV-1 carriers. Int J Cancer.

[7] Ibrahim F, Fiette L, Gessain A, Buisson N, de-The G, Bomford R (1994). Infection of rats with human T-cell leukemia virus type-I: susceptibility of inbred strains, antibody response and provirus location. Int J Cancer.

[8] Fang J, Kushida S, Feng R, Tanaka M, Kawamura T, Abe H, Maeda N, Onobori M, Hori M, Uchida K, Miwa M (1998). Transmission of human T-cell leukemia virus type 1 to mice. J Virol.

[9] Feng R, Kabayama A, Uchida K, Hoshino H, Miwa M (2001). Cell-free entry of human T-cell leukemia virus type 1 to mouse cells. Jpn J Cancer Res.

[10] Kushida S, Maeda N, Fang J, Uchida K, Miwa M (1997). Establishment of HTLV-1 carrier mice by injection with HTLV-1-producing T cells. Leukemia.

[11] Yoshida A, Tanaka R, Murakami T, Takahashi Y, Koyanagi Y, Nakamura M, Ito M, Yamamoto N, Tanaka Y (2003). Induction of protective immune responses against R5 human immunodeficiency virus type 1 (HIV-1) infection in hu-PBL-SCID mice by intrasplenic immunization with HIV-1-pulsed dendritic cells: possible involvement of a novel factor of human CD4(+) T-cell origin. J Virol.

[12] Sondergaard H, Kvist PH, Haase C (2013). Human T cells depend on functional calcineurin, tumour necrosis factor-alpha and CD80/CD86 for expansion and activation in mice. Clin Exp Immunol.

[13] Etoh K, Tamiya S, Yamaguchi K, Okayama A, Tsubouchi H, Ideta T, Mueller N, Takatsuki K, Matsuoka M (1997). Persistent clonal proliferation of human T-lymphotropic virus type I-infected cells in vivo. Cancer Res.

[14] Miyazato P, Yasunaga J, Taniguchi Y, Koyanagi Y, Mitsuya H, Matsuoka M (2006). De novo human T-cell leukemia virus type 1 infection of human lymphocytes in NOD-SCID, common gamma-chain knockout mice. J Virol.

[15] Saito M, Matsuzaki T, Satou Y, Yasunaga J, Saito K, Arimura K, Matsuoka M, Ohara Y (2009). In vivo expression of the HBZ gene of HTLV-1 correlates with proviral load, inflammatory markers and disease severity in HTLV-1 associated myelopathy/tropical spastic paraparesis (HAM/TSP). Retrovirology.

[16] Hanon E, Hall S, Taylor GP, Saito M, Davis R, Tanaka Y, Usuku K, Osame M, Weber JN, Bangham CR (2000). Abundant tax protein expression in CD4+ T cells infected with human T-cell lymphotropic virus type I (HTLV-I) is prevented by cytotoxic T lymphocytes. Blood.

[17] Yoshie O, Fujisawa R, Nakayama T, Harasawa H, Tago H, Izawa D, Hieshima K, Tatsumi Y, Matsushima K, Hasegawa H, Kanamaru A, Kamihira S, Yamada Y (2002). Frequent expression of CCR4 in adult T-cell leukemia and human T-cell leukemia virus type 1-transformed T cells. Blood.

[18] Yamano Y, Araya N, Sato T, Utsunomiya A, Azakami K, Hasegawa D, Izumi T, Fujita H, Aratani S, Yagishita N, Fujii R, Nishioka K, Jacobson S, Nakajima T (2009). Abnormally high levels of virus-infected IFN-gamma + CCR4+ CD4+ CD25+ T cells in a retrovirus-associated neuroinflammatory disorder. PLoS One.

[19] Kinpara S, Hasegawa A, Utsunomiya A, Nishitsuji H, Furukawa H, Masuda T, Kannagi M (2009). Stromal cell-mediated suppression of human T-cell leukemia virus type 1 expression in vitro and in vivo by type I interferon. J Virol.

[20] Fan N, Gavalchin J, Paul B, Wells KH, Lane MJ, Poiesz BJ (1992). Infection of peripheral blood mononuclear cells and cell lines by cell-free human T-cell lymphoma/leukemia virus type I. J Clin Microbiol.

[21] Igakura T, Stinchcombe JC, Goon PK, Taylor GP, Weber JN, Griffiths GM, Tanaka Y, Osame M, Bangham CR (2003). Spread of HTLV-I between lymphocytes by virus-induced polarization of the cytoskeleton. Science.

[22] Baba E, Nakamura M, Tanaka Y, Kuroki M, Itoyama Y, Nakano S, Niho Y (1993). Multiple neutralizing B-cell epitopes of human T-cell leukemia virus type 1 (HTLV-1) identified by human monoclonal antibodies. A basis for the design of an HTLV-1 peptide vaccine. J Immunol.

[23] Tanaka Y, Tanaka R, Terada E, Koyanagi Y, Miyano-Kurosaki N, Yamamoto N, Baba E, Nakamura M, Shida H (1994). Induction of antibody responses that neutralize human T-cell leukemia virus type I infection in vitro and in vivo by peptide immunization. J Virol.

[24] Akari H, Suzuki T, Ikeda K, Hoshino H, Tomono T, Murotsuka T, Terao K, Ito H, Yoshikawa Y (1997). Prophylaxis of experimental HTLV-I infection in cynomolgus monkeys by passive immunization. Vaccine.

[25] Kataoka R, Takehara N, Iwahara Y, Sawada T, Ohtsuki Y, Dawei Y, Hoshino H, Miyoshi I (1990). Transmission of HTLV-I by blood transfusion and its prevention by passive immunization in rabbits. Blood.

[26] Sawada T, Iwahara Y, Ishii K, Taguchi H, Hoshino H, Miyoshi I (1991). Immunoglobulin prophylaxis against milkborne transmission of human T cell leukemia virus type I in rabbits. J Infect Dis.

[27] Tanaka Y, Takahashi Y, Tanaka R, Kodama A, Fujii H, Hasegawa A, Kannagi M, Ansari AA, Saito M (2014). Elimination of human T cell leukemia virus type-1-infected cells by neutralizing and antibody-dependent cellular cytotoxicity-inducing antibodies against human T cell leukemia virus type-1 envelope gp46. AIDS Res Hum Retroviruses.

[28] Saito M, Tanaka R, Arishima S, Matsuzaki T, Ishihara S, Tokashiki T, Ohya Y, Takashima H, Umehara F, Izumo S, Tanaka Y (2013). Increased expression of OX40 is associated with progressive disease in patients with HTLV-1-associated myelopathy/tropical spastic paraparesis. Retrovirology.

[29] Durandy A, Kaveri SV, Kuijpers TW, Basta M, Miescher S, Ravetch JV, Rieben R (2009). Intravenous immunoglobulins–understanding properties and mechanisms. Clin Exp Immunol.

