# Supplementary material for: The neutralizing function of the anti-HTLV-1 antibody is essential in preventing in vivo transmission of HTLV-1 to human T cells in NOD-SCID/γcnull (NOG) mice
Source: Retrovirology. 2014 Aug 28;11:74. doi: 10.1186/s12977-014-0074-z (PMC4180130; doi:10.1186/s12977-014-0074-z)
Supplement: Additional file 1: Table S1. — Sequences flanking the integration site of HTLV-1 provirus in ILT-M1 cell and primer sequences used for integration site-specific PCR. [file 12977_2014_74_MOESM1_ESM.doc]

**Supplemental Table 1**

**Sequences flanking the integration site of HTLV-1 provirus in ILT-M1 cell and primer sequences used for integration site-specific PCR.**

| 5’-genomic region | 5’-LTR |  | 3’-LTR | 3’-genomic region |
| --- | --- | --- | --- | --- |
| TGCTTTGTCATCTGTGCGTTCAGTTCA**TGACAATGACCATGAGCCCCAAATATC** | |  | **TCCAGGAGAGAAACTTAGTACACA**AGTTCACAGAGTTTCACCTTTCTCTTCA | |
| Forward Primer for the 5’-genomic region | Reverse Primer for the 5’-LTR |  | Forward Primer for the 3’-LTR | Reverse Primer for the 3’-genomic region |
| 5’-TGCAGATTTCAAGCGCTTCTAGG-3’ | 5’-TTAGTCTGGGCCCTGACCTTTTCA-3’ |  | 5’-CAACTCTACGTCTTTGTTTCGT-3’ | 5’-GTAAATGAGAAATCCCGCTTCCA-3’ |

Flanking sequences of 5’- and 3’-LTR were determined by inverse-PCR.

HTLV-1 proviral sequences are shown in boldface.
