# Supplementary material for: The neutralizing function of the anti-HTLV-1 antibody is essential in preventing in vivo transmission of HTLV-1 to human T cells in NOD-SCID/γcnull (NOG) mice
Source: Retrovirology. 2014 Aug 28;11:74. doi: 10.1186/s12977-014-0074-z (PMC4180130; doi:10.1186/s12977-014-0074-z)
Supplement: Additional file 2: Figure S1. — To rule out the possible contamination of residual ILT-M1 cells, inverse PCR amplification was carried out to determine the sequences adjacent to HTLV-1 LTRs (both 3′- and 5′-LTR) using the DNA extracted from ILT-M1 cells, as previously described [13]. Then, integration site-specific PCR was carried out using primer pairs that encompass HTLV-1 LTRs (both 3′ and 5′ LTR) and flanking host sequences (Additional file 1: Table S1). As shown, no integration site specific bands were observed except for ILT-M1 cells, suggesting that the possible contamination of HTLV-1 genome derived from the residual ILT-M1 cells is unlikely. [file 12977_2014_74_MOESM2_ESM.pdf]

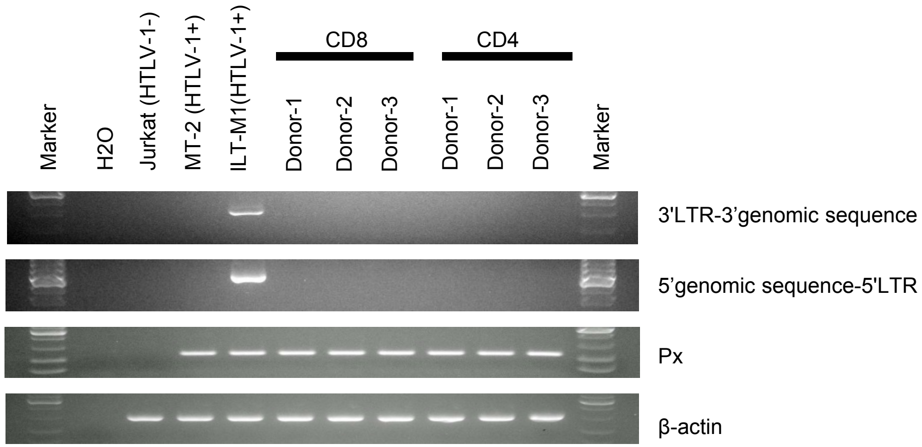

To rule out the possible contamination of residual ILT-M1 cells, inverse PCR amplification was carried out to determine the sequences adjacent to HTLV-1 LTRs (both 3′- and 5′-LTR) using the DNA extracted from ILT-M1 cells, as previously described [13]. Then, integration site-specific PCR was carried out using primer pairs that encompass HTLV-1 LTRs (both 3′ and 5′ LTR) and flanking host sequences (Additional file 1: Table S1). As shown, no integration site specific bands were observed except for ILT-M1 cells, suggesting that the possible contamination of HTLV-1 genome derived from the residual ILT-M1 cells is unlikely.

Supplemental Figure 1
