# Supplementary material for: The neutralizing function of the anti-HTLV-1 antibody is essential in preventing in vivo transmission of HTLV-1 to human T cells in NOD-SCID/γcnull (NOG) mice
Source: Retrovirology. 2014 Aug 28;11:74. doi: 10.1186/s12977-014-0074-z (PMC4180130; doi:10.1186/s12977-014-0074-z)
Supplement: Additional file 3: Figure S2. — Flow cytometric studies showed that the human lymphocytes recovered from mouse spleens express the amount of Tax protein after short-term (16 h) cultivation ex vivo, indicating that the neutralizing anti-Env Ab (clone LAT-27) injection once after PBMC transplantation did not block the in vivo transmission of HTLV-1. [file 12977_2014_74_MOESM3_ESM.pdf]

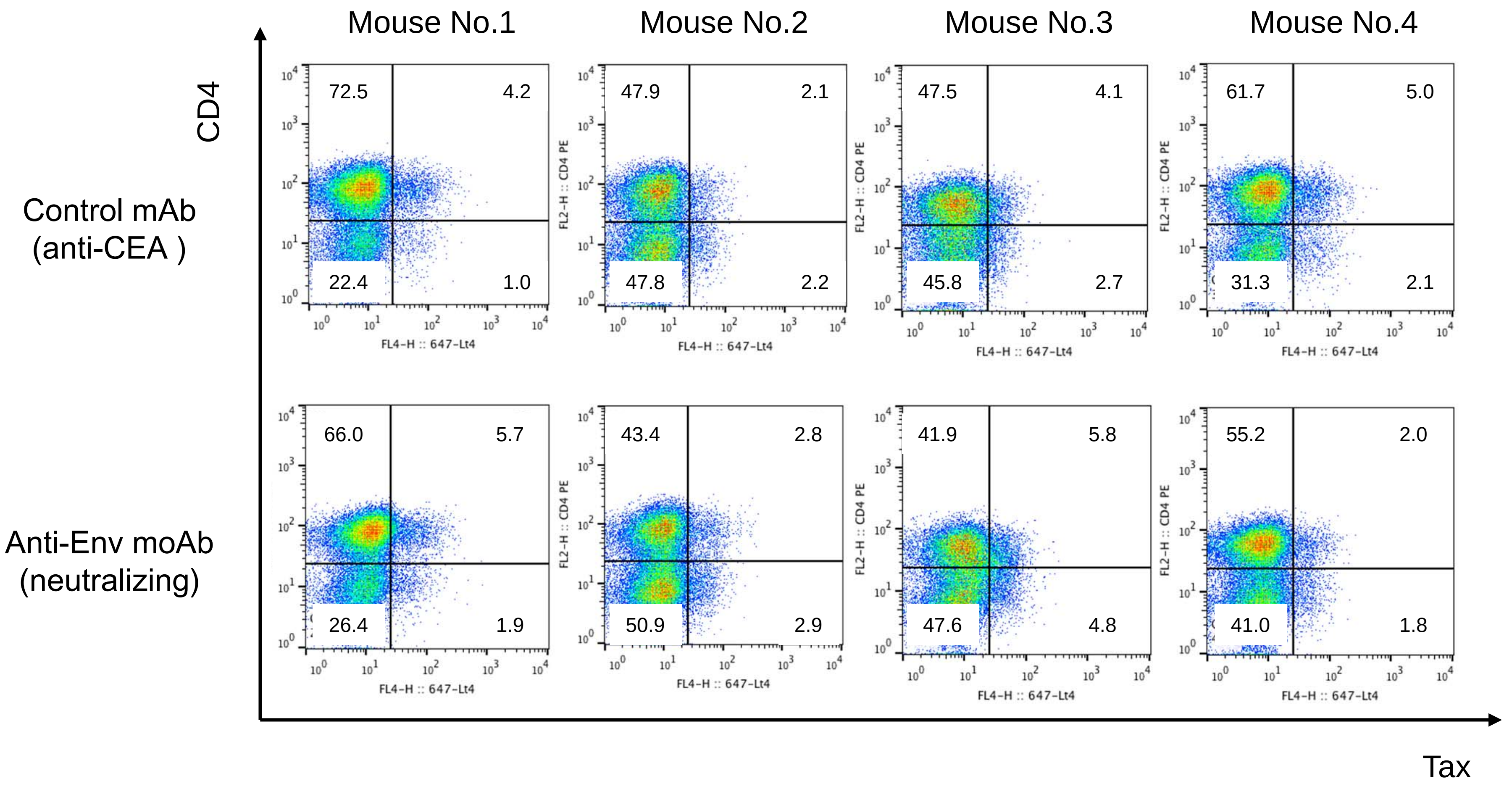

Flow cytometric studies showed that the human lymphocytes recovered from mouse spleens express the amount of Tax protein after short-term (16 h) cultivation ex vivo, indicating that the neutralizing anti-Env Ab (clone LAT-27) injection once after PBMC transplantation did not block the in vivo transmission of HTLV-1.

Supplemental Figure 2
